# Concerns regarding myelofibrosis-type megakaryocyte dysplasia

**DOI:** 10.1038/s41375-024-02160-7

**Published:** 2024-01-23

**Authors:** Yanan Cai, Yuebo Wang, Jing Yang, Zunmin Zhu

**Affiliations:** 1 Department of Hematology, Henan Provincial People’s Hospital, Zhengzhou University, Zhengzhou, China; 2 Clinical Research Service Center, Henan Provincial People’s Hospital, Zhengzhou University, Zhengzhou, China

**Keywords:** Myeloproliferative disease, Diagnosis

## To the Editor:

Myelofibrosis (MF) in Philadelphia chromosome negative myeloproliferative neoplasms (MPNs) mainly presents as primary myelofibrosis (PMF), or can develop from polycythemia vera (post-PV MF) and essential thrombocythemia (post-ET MF). MF is characterized by splenomegaly, anemia, and a constellation of constitutional symptoms with shortened survival and high risk for leukemia transformation. The prominent histological feature of MF in bone marrow biopsies is the presence of megakaryocyte hyperplasia and atypia, which includes dense or loose clustering, frequent endosteal translocation and hyper-chromatic, hypo-lobulated, bulbous or irregularly folded nuclei along with an aberrant nuclear/cytoplasmic ratio according to the revised 2022 WHO criteria [1]. Importantly, these histological features are shared by persons with PMF and persons with post-PV MF and post-ET MF [2].

Based on the aforementioned characteristics of atypical megakaryocytes, Barosi et al. [3] recently introduced the term of myelofibrosis-type megakaryocyte dysplasia (MTMD) to encompass a group of related MPN variants: persons with clonal megakaryocyte dysplasia with normal blood values (CMD-NBV), persons with clonal megakaryocyte dysplasia with isolated thrombocytosis (CMD-IT) and persons with pre-PMF or overt-PMF. The definition of MTMD originated from megakaryocyte morphology and provided a novel perspective for understanding the clinicopathology of MPNs. However, there are still several questions that require further elucidation.

Firstly, persons with CMD-NBV were typically diagnosed in the context of recent or past vein thrombotic event in atypical site or unexplained arterial thrombosis with or without driver mutations [3]. However, a question arises regarding how to approach bone marrow biopsy in persons who do not exhibit abnormal blood values nor suffer thrombosis or driver mutations. This raises ethical concerns and necessitates an evaluation of the true prevalence of this clinical entity. However, there are still several circumstances that should raise concerns about the possible existence of CMD-NBV. One such circumstance is the occurrence of idiopathic splanchnic vein thromboses (SVTs), which include thrombosis in the portal, hepatic, mesenteric, and splenic veins. These SVTs have been found to precede the diagnosis of MPNs and have also been associated with clonal hematopoiesis of indeterminate potential (CHIP) by *DNMT3A* mutations [4, 5]. Additionally, *JAK2V617F*-mutated CHIP has been observed in individuals with arterial thrombosis, such as ischemic stroke [6]. It is possible that CHIP may progressively expand over time and eventually develop into overt MPNs. Even without progression to MPNs, CHIP is associated with a higher risk of recurrent venous and arterial thrombosis, type 2 diabetes and atherosclerotic cardiovascular disease [7]. Therefore, in individuals with idiopathic SVTs or unexplained ischemic stroke, it is not only necessary but also feasible to perform next-generation sequencing on peripheral blood samples to screen for clonal hematopoiesis markers, including driver mutations and non-driver mutations. The presence of at least one of these markers would then warrant further examination of the bone marrow, as proposed by Barosi et al. [8]. This diagnostic strategy would enable the early identification of at least a subgroup of individuals with occult CMD-NBV. However, it is important to note that implementing this strategy may come at the expense of diagnostic profitability. Secondly, it remains an open question whether there exists a continuous biological spectrum from CMD-NBV or CMD-IT to pre-PMF or overt-PMF. Further investigations are needed to elucidate the lateral evolutionary trajectory among these entities in larger cohorts. This includes determining whether they maintain a steady state over a long period or follow an evolutionary pattern step by step or by leaps and bounds. Thirdly, it is megakaryocyte morphology that has been emphasized by MTMD on histological grounds [3]. The development from PV and ET toward post-PV MF and post-ET MF represents a biological continuum with synchronous occurrence of megakaryocyte hyperplasia and atypia, resembling the histomorphologic features of MTMD [2]. Although neither post-PV MF nor post-ET MF fulfill the criteria of MTMD which excludes the initial diagnosis of PV or ET, there is a suspicion that in the evolutionary process whether there exist intermediate stages which could be defined by morphological parameters such as histological megakaryocyte characteristics and myelofibrosis grade. In September 2023, a 39-year-old male person was admitted to our center due to abdominal distension since July 2022. The person had previously been diagnosed with PV in September 2021, characterized by a hemoglobin level of 182 g/l and the presence of *JAK2V617F* mutation, but without splenomegaly. The initial bone marrow examination revealed pan-myelosis and megakaryocytic hyperplasia with pleiomorphism, consistent with the typical marrow features of PV. No megakaryocyte atypia, such as hypo-lobulated or bulbous nuclei and maturation defects, was recorded. Reticulin deposition was graded as 0 according to the European consensus. The person was prescribed oral hydroxyurea, although adherence to the medication was irregular. Upon admission, the hemoglobin level was measured at 172 g/l, and spleen was palpable 3 centimeters below the rib margin. Next-generation sequencing confirmed the presence of the *JAK2V617F* mutation with a variant allele frequency of 87.5%. A reevaluation of the bone marrow biopsy revealed trilineage hyperplasia with grade 1 bone marrow fibrosis. However, notable atypical megakaryocytes with dense clustering and hypo-lobulated and bulbous nuclei were observed, analogous to the typical histological features of PMF. This raises the question of how to diagnose persons who fulfill WHO criteria for post-PV MF and post-ET MF other than degree of fibrosis but with megakaryocyte atypia. Gowin et al. [9] noted that MF-1 persons with PV or ET shared similar clinical characteristics with PMF persons, indicating that the sole consideration of fibrosis grade has limitations in defining disease progression in PV and ET. Moreover, integration of fibrosis degree with megakaryocyte morphology could refine disease classification boundaries [10]. Therefore, we use MTMD for reference and propose that PV or ET persons could be divided into four categories according to fibrosis grade and megakaryocyte features: persons with MF <2 with or without MTMD-like megakaryocytes and persons with MF ≥ 2 with or without MTMD-like megakaryocytes. The presence of MTMD-like megakaryocytes in PV or ET persons with MF <2 might indicate an intermediate biological status toward overt MF from a morphological perspective as observed in our case. This classification system might improve early detection of PV or ET persons at risk of progression to myelofibrosis, thereby favoring early therapeutic interventions to interrupt disease development. Due to the relatively small population of this subgroup persons, multi-center collaboration is warranted to verify this hypothesis. Lastly, whether persons with CMD-NBV or CMD-IT should adopt a watch-and-wait approach or could benefit from interventions such as hydroxyurea, interferon α or JAK inhibitors like Ruxolitinib remains an unsettled issue. Determining the optimal timing for intervention and the appropriate treatment strategies require in-depth discussions.
